# Relationship between L-DOPA-induced reduction in motor and exploratory activity and degree of DAT binding in the rat

**DOI:** 10.3389/fnbeh.2014.00431

**Published:** 2014-12-17

**Authors:** Susanne Nikolaus, Markus Beu, Angelica Maria De Souza Silva, Joseph P. Huston, Hubertus Hautzel, Owen Y. Chao, Christina Antke, Hans-Wilhelm Müller

**Affiliations:** ^1^Clinic of Nuclear Medicine, University Hospital DüsseldorfDüsseldorf, Germany; ^2^Center for Behavioural Neuroscience, Institute of Experimental Psychology, Heinrich-Heine UniversityDüsseldorf, Germany

**Keywords:** dopamine transporter, L-DOPA methylester, motor behavior, exploratory behavior, habituation, time-behavior curves, small animal SPECT

## Abstract

**Purpose**: The present study assessed the influence of L-DOPA administration on neostriatal dopamine (DA) transporter (DAT) binding in relation to motor and exploratory behaviors in the rat.

**Methods**: Rats received injections of 5 mg/kg L-DOPA, 10 mg/kg L-DOPA or vehicle. Motor and exploratory behaviors were assessed for 30 min in an open field prior to administration of [^123^I]FP-CIT. Dopamine transporter binding was measured with small animal single-photon emission computed tomography (SPECT) 2 h after radioligand administration for 60 min.

**Results**: Both L-DOPA doses significantly reduced DAT binding and led to significantly less head-shoulder motility and more sitting relative to vehicle. Moreover, 10 mg/kg L-DOPA induced less distance traveled and ambulation than 5 mg/kg L-DOPA. Analysis of time-behavior (t-b) curves showed that L-DOPA-treated animals relative to vehicle exhibited (1) a faster rate of increase in duration of sitting; (2) a slower rate of increase in duration of head-shoulder motility; and (3) a slower rate of decrease in frequency of head-shoulder motility.

**Conclusions**: The reductions of striatal DAT binding after L-DOPA challenges reflected elevated concentrations of synaptic DA. L-DOPA-treated animals showed less head-shoulder motility and more sitting than vehicle-treated animals, indicating an association between less behavioral activity and increased availability of striatal DA. The faster increase of sitting duration to a higher final level and the slower increase of head-shoulder motility to a lower final level relative to controls may be interpreted in terms on behavioral habituation to a novel environment.

## Introduction

Presynaptic monoamine transporters such as the dopamine transporter (DAT) regulate neurotransmitter concentrations available for receptor binding at the postsynaptic site. Deficiencies of DAT function play a major role in the pathophysiology of psychiatric disorders (for review see, e.g., Nikolaus et al., 2009b, 2010, 2012, 2014) and a variety of neurological conditions (for review see, e.g., Nikolaus et al., 2009a) including idiopathic and early-onset Parkinson’s disease (PD) and the “Parkinson Plus” syndromes multiple system atrophy and progressive supranuclear palsy. In idiopathic PD and “Parkinson Plus” syndromes, DAT function is affected to different degrees, while it is unimpaired in essential tremor (e.g., Plotkin et al., 2005). Therefore, DAT imaging with positron emission tomography (PET) or single-photon emission computed tomography (SPECT) has become an important tool to differentiate between movement disorders.

The treatment of choice for idiopathic PD is L-3,4-dihydroxyphenylalanine (L-DOPA), which passes the blood-brain barrier via a saturable transporter and is converted to dopamine (DA) in the presynaptic terminal by aromatic L-amino acid decarboxylase (for review see Okereke, 2002). Initially, the increased availability of DA compensates for the decline of DA synthesis, DA storage and DAT binding, which are characteristic for idiopathic PD. With extended L-DOPA treatment, however, inhibitory feedback mechanisms at the presynaptic terminal lead to a reduction of endogenous DA formation, resulting in an aggravation of DAergic depletion. Common features of long-term treatment with L-DOPA, therefore, are dyskinesias, on-off phenomenon and wearing-off phenomenon (for review see Cenci et al., 2011).

As of yet, the displaceability of DAT radioligands by endogenous DA in *in vivo* imaging is still a matter of debate. The process of nigrostriatal neurodegeneration leads to a progressive reduction of DAT binding sites and diminishes the capacity to synthesize and release DA contingent on administration of the precursor molecule. Thus, it is likely that the extent of neurodegeneration determines whether a reduction of radioligand binding occurs after treatment with L-DOPA. If DAT binding is assessed in less severe cases in baseline and after L-DOPA challenge, it may be assumed that DA is released into the synaptic cleft in relation to the extent of striatal lesion leading to competition with the exogenous radioligand and a reduction of DAT binding compared to baseline. Accordingly, acute and chronic treatment with clinical and higher L-DOPA doses has been shown to significantly reduce radioligand binding to the DAT in PD patients with lower disease severity (Guttman et al., 2001), in healthy rats (Dresel et al., 1998; Nikolaus et al., 2011b, 2013) and in the intact striata of unilaterally 6-hydroxydopamine-lesioned animals (Sossi et al., 2010).

In rats, L-DOPA is known to affect motor behaviors, leading to decreased activity in mature animals after doses of 12.5–50 mg/kg (McDevitt and Setler, 1981) and to increased activity in immature animals after 150 mg/kg (Grigoriadis et al., 1996). In a previous study (Nikolaus et al., 2013), we presented preliminary *in vivo* evidence for an association between neostriatal DAT binding and parameters of motor and exploratory behavior after treatment with L-DOPA doses of 5 and 10 mg/kg. In the present study, we expand on these findings with a modified design that allows comparisons between groups rather than between treatment and baseline and a more differentiated approach to the assessment of behaviors. Generally, drug effects on behavior are assessed by evaluating the relationship between dose and magnitude of behavioral response. Since, however, compounds acting on the DAergic system are also likely to influence behavior over time, our rationale was to gain information on the temporal dynamics of behavioral alterations. For this purpose, we plotted the individual behaviors against the time post-injection and determined time-behavior (t-b) curves by deriving suitable mathematical models for the vehicle-treated animals. These models were fit to the behavioral data of the animals treated with 5 or 10 mg/kg L-DOPA, and the resulting t-b curves were compared between treatment groups.

Furthermore, we aimed to investigate, whether alterations of behaviors can be related to changes in magnitude of DAT binding relative to controls. Therefore, motor and exploratory behaviors were correlated with DAT binding and subjected to a multivariate principal component analysis (PCA) and a cluster analysis (CA).

## Materials and methods

### Animals

The present study employed a total of 52 adult male Wistar rats (TVA, Heinrich-Heine University, Düsseldorf, Germany), weighing 395 ± 49 g (mean ± SD). Data obtained on 19 animals out of this pool were reported as a pilot study (Nikolaus et al., 2013). Dopamine transporter binding and behavior were jointly assessed after treatment with 5 mg/kg L-DOPA (*n* = 15), 10 mg/kg L-DOPA (*n* = 14) and vehicle (0.9% saline; *n* = 16). Seven animals merely underwent behavioral assessment (5 mg/kg L-DOPA, *n* = 3; 10 mg/kg L-DOPA, *n* = 3; saline, *n* = 1). Rats were maintained in standard macrolon cages (590 × 380 × 200 mm; 3 animals per cage) in a climate cabinet (Scantainer, Scanbur BK, Karslunde, Denmark; temperature, 20°C; air humidity, 70%) with an artificial ligh-dark cycle (lights on ar 6:00 a.m.; lights off at 6:00 p.m.) and food and water freely available. The study was approved by the regional authority and carried out in accordance with the “Principles of laboratory animal care” (NIH publication No. 86–23, revised 1985) and the German Law on the Protection of Animals.

### SPECT camera

The small animal tomograph (“TierSPECT”) was described in detail elsewhere (Schramm et al., 2000). Tomographic resolutions (FWHM) are to 3.4 and 2.8 mm for ^123^I and ^99m^Tc, respectively, while sensitivities amount to 16 (^123^I) and 22 cps/MBq (^99m^Tc). A low-energy ultra-high-resolution parallel-hole collimator (LEUHR, 37 × 1 × 0.2 mm^3^) was mounted in front of the detector head. Data were acquired in a 128 × 128 matrix with a pixel width and a slice thickness of ≈ 0.664 mm, respectively. Data were acquired for 60 min in a step-and-shoot mode over a circular orbit in angular steps of 6° (60 projections, 60 s/projection) using a 65 mm radius of rotation. Reconstruction was performed with an iterative ordered-subset-expectation-maximization algorithm (3 iterations, 4 subsets/iteration). No post-filtering procedure was applied. An attenuation correction of 0.11 cm^−1^ was implemented for both ^123^I and ^99m^Tc assuming a uniformly attenuating medium.

### DAT imaging studies

Dopamine transporter binding was assessed after challenge with L-DOPA methylester (Sigma-Aldrich, Taufkirchen, Germany; dose, 5 or 10 mg/kg; concentrations, 5 or 10 mg/ml) plus benserazide (Sigma-Aldrich, Taufkirchen, Germany; dose, 10 mg/kg; concentration, 10 mg/ml) or vehicle (0.9% saline; B. Braun Melsungen AG, Melsungen, dose: 1 ml/kg). Benserazide is peripherally-acting aromatic L-amino acid decarboxylase inhibitor, which is applied in order to prevent the metabolization of L-DOPA before it passes the blood-brain barrier (Shen et al., 2003).

Challenges were applied intraperitoneally (i.p.) 30 min prior to radioligand application, since maximum striatal DA concentrations are reached at 30 min after i.p. L-DOPA and remain stable for approximately 2 h (Shen et al., 2003). L-3,4-dihydroxyphenylalanine and benserazide were administed simultaneously, since previous investigations had shown that pre-treatment with benserazide up to 3 h prior to L-DOPA administration did not alter motor responses to L-DOPA compared to simultaneous application of both compounds (Tayarani-Binazir et al., 2010). Thirty minutes after L-DOPA plus benserazide or saline, animals received i.p. injections of 0.9 ml/kg ketaminehydrochloride (Ketavet®, Pharmacia GmbH, Erlangen, Germany; concentration, 100 mg/ml) and 0.4 ml/kg xylazinehydrochloride (Rompun® Bayer, Leverkusen, Germany; concentration, 2%). Then 27 ± 4 MBq [^123^I]-FP-CIT (DATSCAN, GE Healthcare, München, Germany; concentration range, 0.07–0.13 μg/ml, specific activity range, 2.5–4.5 × 10^14^ Bq/mmol at reference time) were applied into the lateral tail vein using a winged infusion needle set. The tube was rinsed with 1 ml 0.9% saline amounting to a total injection volume of 1.3 ml.

Since the equilibrium of [^123^I]FP-CIT binding is reached at 2 h post-injection with the ratio of specific to non-specific striatal uptake remaining stable over the following 4 h (Booi et al., 1997), SPECT measurements were started 2 h after radioligand application. Since SPECT measurements were conducted over 1 h, animals were kept under anesthesia for a total of 3 h.

### Behavioral studies

Immediately after the injection of 5 mg/kg L-DOPA, 10 mg/kg L-DOPA or vehicle rats were placed into the center of a cage with a topunit equipped with light-emitting diodes and a charge-coupled device (CCD) camera (Phenotyper®, Noldus Information Technology, Wageningen, Netherlands; open field dimensions, 45 cm × 45 cm × 56 cm). Durations (s) and frequencies (n) of motor and exploratory behaviors were rated blindly in blocks of 5 min for a total of 30 min using EthoVision XT (Noldus Information Technology, Wageningen, Netherlands). Rated behaviors were: (A) ambulation as a measure of motor activity; (B) sitting without any motion; (C) rearing (freely standing or leaning against the wall) as a measure of both motor activity and non-selective attention according to Aspide et al. (1998); (D) head-shoulder motility (movements of head and shoulders while the animal was sitting), a parameter recently introduced as a further measure of motor activity and non-selective attention (Nikolaus et al., 2013); (E) grooming (fur, paw and claw licking, scratching). In addition, based on the dislocation of the animal’s center point EthoVision XT automatically determined the distance in centimeters traveled by the rat. Following the behavioral trials, animals were anesthetized and administered [^123^I]FP-CIT as described above.

### Evaluation of DAT imaging studies

Imaging data were evaluated using the Multi-Purpose-Imaging-Tool (MPI-Tool V3.29, Advanced Tomo Vision GmbH, Kerpen, Germany) as previously described (e.g., Nikolaus et al., 2007, 2011a, 2013). Briefly, maximum striatal count rates (counts/pixel) were determined on coronal slices by defining a circular region covering an area of 1.5 mm^2^, which comprised a total of 11 pixels. On the same slices, cerebellar reference count rates (counts/pixel) were obtained in an elliptic region (area, 7 mm^2^ comprising 53 pixels) approximately 15 mm posterior to the frontal cortex corresponding anatomically to the rat cerebellum. Left and right striatal counts rates were averaged. The equilibrium ratios of the distribution volumes of the specifically and the non-specifically bound compartment (V_3_” = V_T_(striatum)/V_T_(cerebellum)–1) were computed as estimates for the binding potentials (Laruelle et al., 1994).

### Statistical analysis

#### DAT imaging studies

Distributions of binding data were assessed with the non-parametric Kolmogorov-Smirnov test (*α* = 0.05). In each pre-treatment condition, V_3_” values as well as cerebellar count rates were found to be normally distributed (0.313 ≤ *p* ≤ 0.989). Striatal V_3_” values and cerebellar radioactivity count rates obtained after 5 mg/kg L-DOPA, 10 mg/kg L-DOPA or vehicle were compared with the parametric independetnt *t* test (two-sided, *α* = 0.0167 after Bonferroni correction for multiple comparisons). Calculations were performed using IBM SPSS Statistics 22.

#### Behavioral studies

Distributions of behavioral data (traveled distance [cm], duration [s] and frequency [n] of ambulation, sitting, rearing, head-shoulder motility and grooming in 5-min bins were assessed with the non-parametric Kolmogorov-Smirnov test (*α* = 0.05). Since in all pre-treatment conditions, the majority of behavioral parameters was not found to be normally distributed (0.2 ≤ *p* ≤ 0.0001), behaviors in each 5-min time bin were compared between groups using the non-parametric Mann-Whitney *U* test (two-sided, *α* = 0.0167 after Bonferroni correction for multiple comparisons). Calculations were performed using IBM SPSS Statistics 22.

The medians of the individual behavioral parameters obtained after vehicle (Y-axis: traveled distance, duration and frequency of ambulation, sitting, rearing, head-shoulder motility or grooming) were plotted against the end-points of the individual time frames (X-axis). Upon visual inspection of the data, the following mathematical models were fit to the individual behavioral parameters, using either non-linear or linear regression analysis with the regression coefficient (*R*^2^) as a measure for the goodness of fit: (1) *traveled distance*, exponential function (*y*(*t*) = a * exp (−K * *x*) + plateau with *a*, value at the time *t*; K, rate constant; *t*, time); *R*^2^ = 0.906; (2) *ambulation duration*; exponential function; *R*^2^ = 0.889; (3) *ambulation frequency*; exponential function; *R*^2^ = 0.946; (4) *sitting duration*; linear function (*y* = *ax* + *b* with *a*, slope and *b*, *y*-intercept); *R*^2^ = 0.778; (5) *sitting frequency*; quadratic function (*y* = *a* + *bx* + *cx*^2^ with *a*, absolute term; *bx*, linear term; *cx*^2^, quadratic term); *R*^2^ = 0.636; (6) *rearing duration*; exponential function; *R*^2^ = 0.858; (7) *rearing frequency*; exponential function; *R*^2^ = 0.957; (8) *duration of head-shoulder motility*; quadratic function; *R*^2^ = 0.968; (9) *frequency of head-shoulder motility*; linear function; *R*^2^ = 0.925).

The same models were fit to the behavioral data obtained after 5 and 10 mg/kg L-DOPA with *R*^2^ values respectively of 0.997 and 0.991 (traveled distance), 0.985 and 0.975 (ambulation duration), 0.991 (ambulation frequency), 0.945 and 0.782 (sitting duration), 0.786 and 0.532 (sitting frequency), 0.968 and 0.988 (rearing duration), 0.992 and 0.976 (rearing frequency), 0.879 and 0.477 (duration of head-shoulder motility) and 0.695 and 0.84 (frequency of head-shoulder motility). Due to the low expression of both grooming duration and grooming frequency, the grooming data could not be reasonably fit to any mathematical model. Curve fittings were performed using GraphPad Prism (version 3.0 for Windows, GraphPad Software, San Diego, USA). T-b curves were compared between treatment groups using the *F* test (*α* = 0.0167 after Bonferroni correction for multiple comparisons).

Correlation analysis, PCA and CA are different methods providing information on the interrelation of variables. While correlation analysis describes the cross-covariance between two sets of data, PCA identifies principal components accounting for the variability of the data set. Cluster analysis in turn, groups a set of variables in such a way that variables in the same group (cluster) are more similar to each other than to those in other groups. In order to gauge the extent of association between DAT binding and motor/exploratory parameters, Spearman rank correlation coefficients (*r*) were computed (*α* = 0.05) for V_3_” values and behavioral data (traveled distance, duration and frequency of ambulation, sitting, rearing, head-shoulder motility and grooming in min 1–5, 6–10, 11–15, 16–20, 21–25, 26–30 and 1–30). Calculations were performed using IBM SPSS Statistics 22. In addition, data were subjected to a multivariate PCA and to a CA using Rapid Miner (version 4.4., Rapid-I GmbH, Dortmund, Germany).

## Results

### DAT imaging studies

**Figure 1:**
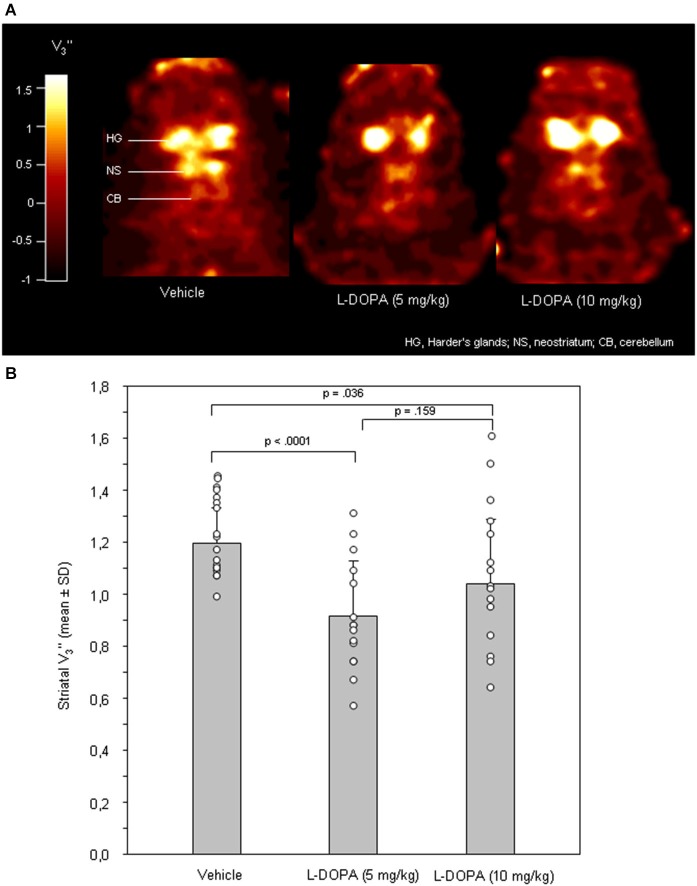
**(A)** Coronal [^123^I]FP-CIT images of rat heads after pre-treatment with vehicle (0.9% saline), 5 mg/kg L-DOPA and 10 mg/kg L-DOPA. The reduction in striatal DAT binding after both L-DOPA doses is clearly visible. All images show V_3_” values; it is understood, that the calculation of V_3_” is only valid for regions of specific radioligand binding such as the rat striatum. Calculations were performed using MATLAB (version 4.2c or version 6, The MathWorks Inc., Novi, USA). **(B)** Striatal equilibrium ratios (V_3_”) after vehicle, 5 mg/kg L-DOPA and after 10 mg/kg L-DOPA. Rendered are means and standard deviations of the means. The circles represent the individual animals. For significant between-group differences the respective *p* values are given (two-tailed independent *t* test, *α* = 0.0167 after Bonferroni correction).

Figure 1A shows characteristic images of [^123^I]FP-CIT accumulations on coronal slices of rat brains after treatment with vehicle, 5 mg/kg L-DOPA or 10mg/kg L-DOPA. Striatal [^123^I]FP-CIT accumulations were markedly reduced following challenge with both 5 and 10 mg/kg L-DOPA.

After vehicle, 5 mg/kg L-DOPA and 10 mg/kg L-DOPA, cerebellar radioactivity concentrations (data not shown) amounted to 881 ± 134 counts/pixel (mean ± standard deviation), 898 ± 295 counts/pixel and 851 ± 311 counts/pixel, respectively. No significant between-group differences were obtained (0.835 ≤ *p* ≤ 0.983).

After application of 5 and 10 mg/kg L-DOPA (Figure 1B), neostriatal V_3_” were 0.91 ± 0.21 and 1.02 ± 0.25, respectively. After vehicle, V_3_” amounted to 1.20 ± 0.14. Significant differences were obtained between 5 mg/kg L-DOPA and vehicle (*p* < 0.0001). For the comparison between 10 mg/kg L-DOPA and vehicle a *p* of 0.036 was obtained, which failed to reach significance after Bonferroni correction. There was no significant difference between 5 and 10 mg/kg L-DOPA (*p* = 0.159).

### Behavioral studies

#### Median differences and t-b curves—traveled distance

**Figure 2:**
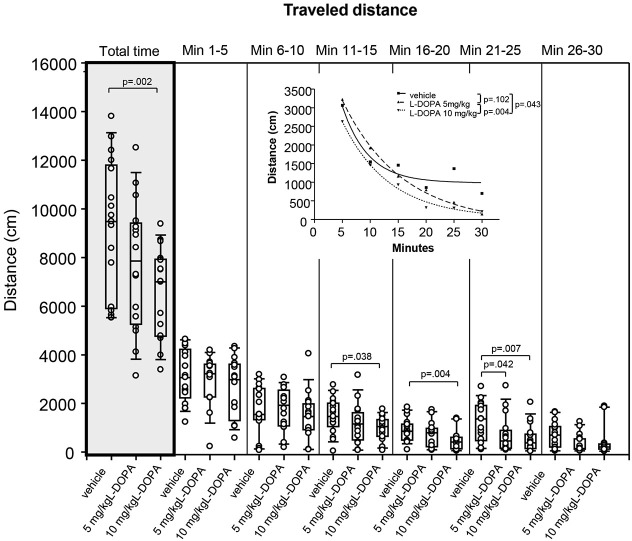
**Traveled distance (cm) after vehicle (0.9% saline), 5 mg/kg L-DOPA and 10 mg/kg L-DOPA**. The figure shows box and whisker plots of median distances traveled during the whole time of testing (gray shade) and in the individual 5-min time bins. 25-/75-percentiles are given in the boxes, while 5-/95-percentiles are represented by the whiskers. The circles represent the individual animals. For significant between-group differences the respective *p* values are given (two-tailed Mann–Whitney *U* test, *α* = 0.0167 after Bonferroni correction). ***Inset***: T-b curves obtained by plotting median values of traveled distances against time and fitting exponential functions (*y*(*t*) = *a* * exp (−*K* * *x*) + plateau with *a*, value at the time *t*; −*K*, rate constant; *t*, time) to these data. For the comparisons between groups (two-tailed *F* test, *α* = 0.0167 after Bonferroni correction) the respective *p* values are given.

After treatment with 10 mg/kg L-DOPA, the median traveled distance (Figure 2) decreased significantly relative to vehicle in min 16–20 (*p* = 0.004) and 21–25 (*p* = 0.007) as well as over the whole trial (*p* = 0.002). Differences between 5 mg/kg L-DOPA and vehicle in min 21–25 (*p* = 0.042) and between 10 mg/kg in min 11–15 (*p* = 0.038) failed to reach significance after Bonferroni correction. Significant between-group differences became evident around min 11.

T-b curves significantly differed between 5 (exponential fit; *a*, 5269 ± 200.2 [mean ± standard error]; *K*, 0.09 ± 0.01; plateau, −98.71 ± 143.8;) and 10 mg/kg L-DOPA (*a*, 4713 ± 405.4; *K*, 0.12 ± 0.02; plateau, 41.02 ± 142.4; *p* = 0.004). Comparison of t-b curves after 10 mg/kg L-DOPA and vehicle (*a*, 6102 ± 3347; *K*, 0.22 ± 0.11; plateau, 981.1 ± 231.1) yielded a *p* of 0.043, and, thus, failed to reach statistical significance after Bonferroni correction. Rate constant and plateau were higher after 10 relative to 5 mg/kg L-DOPA suggesting a *faster* rate of decrease to a higher final level. However, rate constant and plateau were lower than in the vehicle condition, which indicated a *slower rate* of decrease to a lower final level.

#### Ambulation

**Figure 3:**
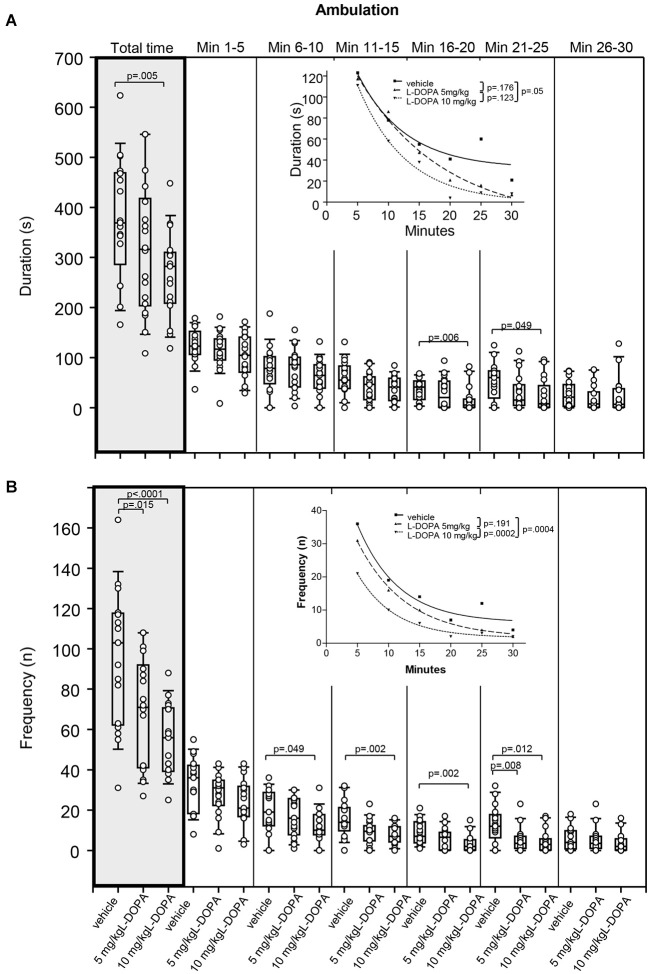
**Ambulation. (A)** Duration (s) and **(B)** frequency (n) after vehicle (0.9% saline), 5 mg/kg L-DOPA and 10 mg/kg L-DOPA. The figure shows box and whisker plots of median ambulation durations during the whole time of testing (gray shade) and in the individual 5-min time bins. 25-/75-percentiles are given in the boxes, while 5-/95-percentiles are represented by the whiskers. The circles represent the individual animals. For significant between-group differences the respective *p* values are given (two-tailed Mann–Whitney *U* test, *α* = 0.0167 after Bonferroni correction). ***Insets***: T-b curves obtained by plotting median values of ambulation durations **(A)** and ambulation frequencies **(B)** against time and fitting exponential functions (*y*(*t*) = *a* * exp (−*K* * *x*) + plateau with *a*, value at the time *t*; −*K*, rate constant; *t*, time) to these data. For the comparisons between treatment groups (two-tailed *F* test, *α* = 0.0167 after Bonferroni correction) the respective *p* values are given.

There was no significant difference in duration of ambulation between animals treated with 5 mg/kg L-DOPA and vehicle (Figure 3A). After 10 mg/kg L-DOPA, rats moved significantly less than after vehicle in min 16–20 (*p* = 0.006) as well as throughout the whole trial (*p* = 0.005). The reduction in ambulation in min 21–25 after 10 mg/kg L-DOPA relative to vehicle failed to reach statistical significance after Bonferroni correction (*p* = 0.049). Significant between-group differences were evident around min 16.

Comparison of t-b curves between 10 mg/kg L-DOPA (exponential fit; *a*, 213.1 ± 34.26; *K*, 0.13 ± 0.04; plateau, −0.46 ± 9.72) and vehicle (*a*, 171.9 ± 61.53; *K*, 0.13 ± 0.08; plateau, 32.06 ± 17.36) yielded a *p* of 0.05, and, thus failed to reach statistical significance after Bonferroni correction. This is underlined by the equality of rate constants. Yet, after L-DOPA, the curve plateau was lower compared to vehicle suggesting a lower final level.

Rats treated with 5 mg/kg L-DOPA displayed a significantly lower ambulation frequency (Figure 3B) compared to vehicle in min 21–25 (*p* = 0.008) as well as throughout the whole trial (*p* = 0.015). After 10 mg/kg L-DOPA, animals moved significantly less frequently compared to the vehicle condition in min 11–15, 16–20 and 21–25 as well as during the whole testing time (0.0001 > *p* ≤ 0.008). Differences between 10 mg/kg L-DOPA and vehicle in min 6–11 (*p* = 0.049) failed to reach statistical significance after Bonferroni correction. Significant between-group differences emerged around min 6.

T-b curves significantly differed between 10 mg/kg L-DOPA (exponential fit; *a*, 44.42 ± 5.52; *K*, 0.17 ± 0.03; plateau, 1.66 ± 0.78) and vehicle (*a*, 63.5 ± 18.19; *K*, 0.15 ± 0.06; plateau, 6.21 ± 3.22; *p* = 0.0005) as well as between 10 and 5 mg/kg L-DOPA (*a*, 55.14 ± 4.03; *K*, 0.13 ± 0.02; plateau, 1.64 ± 1.16; *p* = 0.0005). The curve plateau after 10 mg/kg was lower compared to vehicle, whereas the rate constant was higher. This suggests that after 10 mg/kg L-DOPA, ambulatory frequency decreased at a *faster* rate to a lower final level relative to vehicle. In addition, after 10 mg/kg L-DOPA both rate constant and plateau were higher compared to 5 mg/kg L-DOPA indicating a *faster* rate of increase to a (slightly) higher final level.

#### Sitting

**Figure 4:**
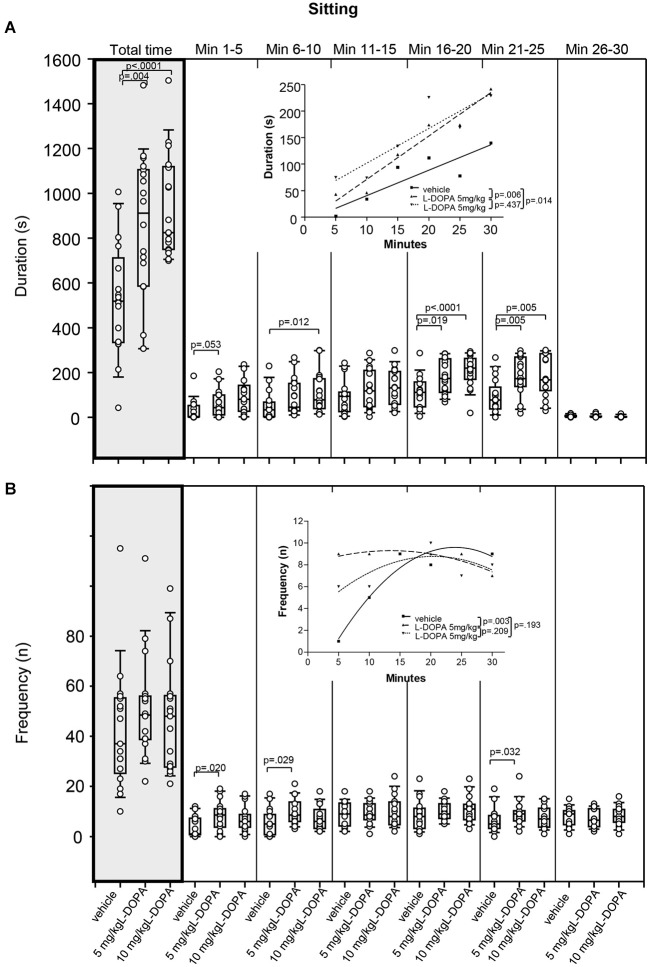
**Sitting. (A)** Duration (s) and **(B)** frequency (n) after vehicle (0.9% saline), 5 mg/kg L-DOPA and 10 mg/kg L-DOPA. The figure shows box and whisker plots of median sitting durations during the whole time of testing (gray shade) and in the individual 5-min time bins. 25-/75-percentiles are given in the boxes, while 25-/95-percentiles are represented by the whiskers. The circles represent the individual animals. For significant between-group differences the respective *p* values are given (two-tailed Mann–Whitney *U* test, *α* = 0.0167 after Bonferroni correction). ***Insets***: T-b curves obtained by plotting median values of sitting durations **(A)** and sitting frequencies **(B)** against time. Linear functions (*y* = *ax* + *b* with *a*, slope and *b*, *y*-intercept) were fitted to the plots of sitting durations, whereas quadrtatic functions (*y* = *a* + *bx* + *cx*^2^ with *a*, absolute term; *bx*, linear term; *cx*^2^, quadratic term) were fitted to the plots of sitting frequencies. For the comparisons between treatment groups (two-tailed *F* test, *α* = 0.0167 after Bonferroni correction) the respective *p* values are given.

Animals treated with 5 mg/kg L-DOPA exhibited significantly longer sitting behavior (Figure 4A) in min 21–25 (*p* = 0.005) as well as throughout the whole trial (*p* = 0.004) compared to rats treated with vehicle. Differences between 5 mg/kg and vehicle in min 1–5 (*p* = 0.053) and min 16–20 (*p* = 0.019) failed to reach significance after Bonferroni correction. After 10 mg/kg L-DOPA, rats sat quietly for a significantly longer time than in the vehicle condition in min 6–10, 16–20 and 21–25 as well as throughout the whole trial (0.0001 ≤ *p* ≤ 0.012). Significant between-group differences were obvious from min 6–10 but temporarily disappeared during the third time frame (min 11–15).

T-b curves significantly differed between 5 mg/kg L-DOPA (linear fit; slope, 8.20 ± 0.99; *y*-intercept, −10.67 ± 19.30) and vehicle (slope, 4.80 ± 0.128; *y*-intercept, −7.33 ± 24.97; *p* = 0.006) as well as between 10 mg/kg L-DOPA (slope, 6.55 ± 1.7; *y*-intercept, 36.47 ± 33.67) and vehicle (*p* = 0.014). The slope of both L-DOPA curves exceeded the slope of the vehicle curve indicating a *faster* rate of increase of sitting duration to a higher final level.

Differences in sitting frequency (Figure 4B) between 5 mg/kg and vehicle in min 1–5 (*p* = 0.020), min 6–10 (*p* = 0.029) and min 21–25 (*p* = 0.032) did not reach significance after Bonferroni correction. There was no significant difference in sitting frequency between animals treated with 10 mg/kg L-DOPA and vehicle.

There was a significant difference between t-b curves after 5 mg/kg L-DOPA (quadratic fit; *a*, 8.0 ± 0.87; *b*, 0.19 ± 0.11; *c*, −0.01 ± 0.003) and vehicle (*a*, −3.77 ± 0.233; *b*, 1.12 ± 0.31; *c*, −0.02 ± 0.01; *p* = 0.003), whereas the t-b curve after 10 mg/kg L-DOPA (*a*, 3.1 ± 0.2.58; *b*, 0.56 ± 0.31; *c*, −0.01 ± 0.01) was neither different from vehicle (*p* = 0.193) nor from 5 mg/kg L-DOPA (*p* = 0.209). After 5 mg/kg L-DOPA, the linear term was lower and the quadratic term was higher compared to vehicle suggesting a *slower* rate of increase of sitting frequency to a higher final level.

#### Rearing

**Figure 5:**
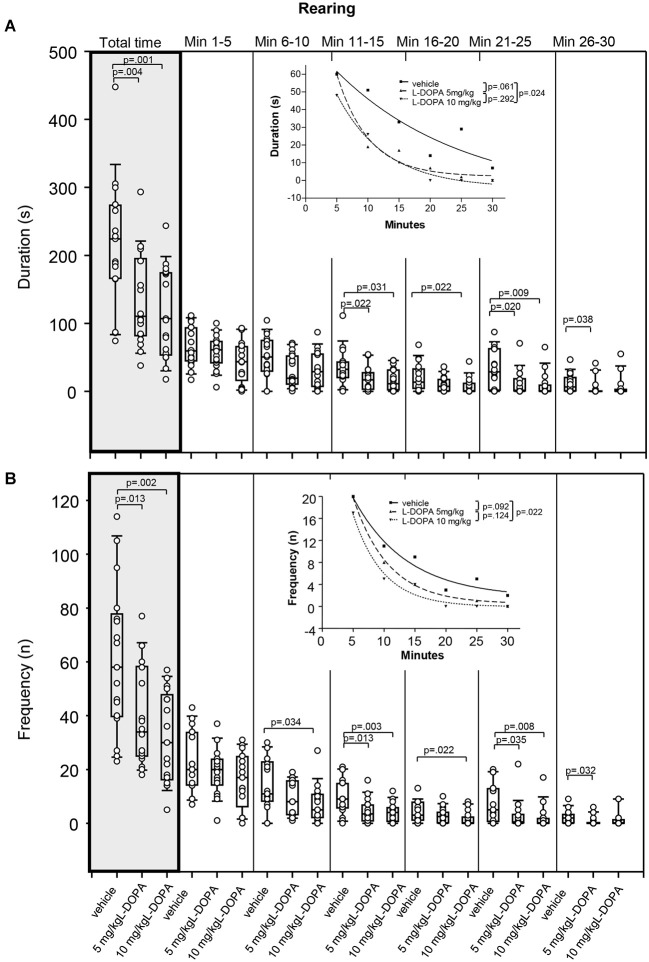
**Rearing. (A)** duration (s) and **(B)** frequency (n) after vehicle (0.9% saline), 5 mg/kg L-DOPA and 10 mg/kg L-DOPA. The figure shows box and whisker plots of median rearing durations during the whole time of testing (gray shade) and in the individual 5-min time bins. 25-/75-percentiles are given in the boxes, while 25-/95-percentiles are represented by the whiskers. The circles represent the individual animals. For significant between-group differences the respective *p* values are given (two-tailed Mann–Whitney *U* test, *α* = 0.0167 after Bonferroni correction). ***Inset***: T-b curves obtained by plotting median values of rearing durations **(A)** and rearing frequencies **(B)** against time and fitting exponential functions (*y*(*t*) = *a* * exp (−*K* * *x*) + plateau with *a*, value at the time *t*; −*K*, rate constant; *t*, time) to these data. For the comparisons between treatment groups (two-tailed *F* test, *α* = 0.0167 after Bonferroni correction) the respective *p* values are given.

After 5 mg/kg L-DOPA, rats reared (Figure 5A) for a significantly shorter time compared to vehicle-treated animals over the whole trial (*p* = 0.004). Differences relative to vehicle in min 11–15 (*p* = 0.022), min 21–25 (*p* = 0.020) and min 26–30 (*p* = 0.038) did not reach significance after Bonferroni correction. Rearing was also significantly shorter after 10 mg/kg L-DOPA compared to vehicle-treated rats in min 21–25 (*p* = 0.009) as well as over the whole trial (*p* = 0.001). Differences relative to vehicle in min 11–15 (*p* = 0.031) and min 16–20 (*p* = 0.022) and min 21–25 failed to reach significance after Bonferroni correction. Significant between-group differences emerged around min 21.

The difference between t-b curves after 10 mg/kg L-DOPA (exponential fit; *a*, 100.9 ± 11.27; *K*, 0.13 ± 0.02; plateau, −4.1 ± 3.17) and vehicle (*a*, 91.47 ± 40.44; *K*, 0.05 ± 0.07; plateau, −9.90 ± 57.18) with a *p* of 0.024 did not reach significance after Bonferroni correction. This also held for the difference between t-b curves after 5 mg/kg L-DOPA (*a*, 153.1 ± 42.17; *K*, 0.19 ± 0.06; plateau, 2.2 ± 3.9 and vehicle (*p* = 0.061). Yet, the rate constants of both L-DOPA curves exceeded those of the vehicle curve indicating a *faster* rate of decrease of rearing, while the higher plateau values implied a higher final level of rearing duration after L-DOPA relative to vehicle.

After 5 mg/kg L-DOPA, rats reared significantly less frequently (Figure 5B) compared to the vehicle condition in min 11–15 (*p* = 0.013) as well as throughout the whole testing time (*p* = 0.013). Differences relative to vehicle in min 21–25 (*p* = 0.035) and min 26–30 (*p* = 0.032) failed to reach statistical significance after Bonferroni correction. After 10 mg/kg L-DOPA, rearing frequency was significantly lower than in vehicle-treated rats in min 11–15 (*p* = 0.003) and min 21–25 (*p* = 0.008) as well as over the whole trial (*p* = 0.002), whereas differences in min 6–10 (*p* = 0.034), min 16–20 (*p* = 0.022) and min 21–25 (*p* = 0.035) failed to reach significance after Bonferroni correction. Significant between-group differences emerged around min 11.

Comparison of t-b curves after 10 mg/kg L-DOPA (exponential fit; *a*, 47.05 ± 11.79; *K*, 0.21 ± 0.05; plateau, −0.047 ± 0.95) and vehicle (*a*, 32.39 ± 6.21; *K*, 0.12 ± 0.04; plateau, 1.64 ± 2.40) yielded a *p* of 0.024, and, thus, after Bonferroni correction did not reach significance. Yet, the higher rate constant after 10 mg/kg L-DOPA suggested a *faster* rate of decrease of rearing frequency, while the lower plateau value indicated a lower final level of rearing frequency compared to vehicle.

#### Head-shoulder motility

**Figure 6:**
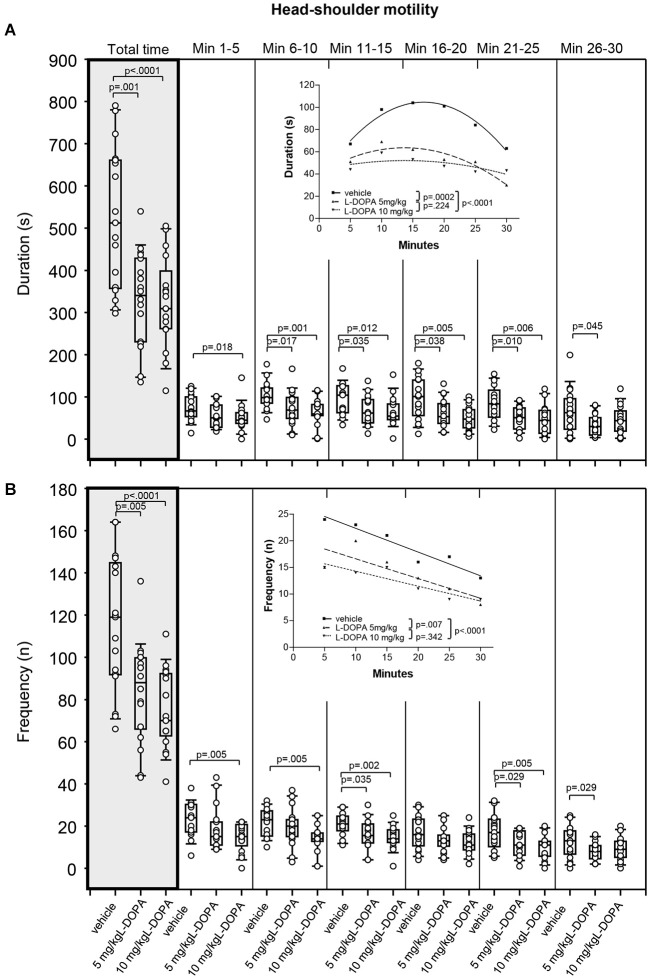
**Head-shoulder motility. (A)** Duration (s) and **(B)** frequency (n) after vehicle (0.9% saline), 5 mg/kg L-DOPA and 10 mg/kg L-DOPA. The figure shows box and whisker plots of median durations of head-shoulder motility during the whole time of testing (gray shade) and in the individual 5-min time bins. 25-/75-percentiles are given in the boxes, while 25-/95-percentiles are represented by the whiskers. The circles represent the individual animals. For significant between-group differences the respective *p* values are given (two-tailed Mann–Whitney *U* test, *α* = 0.0167 after Bonferroni correction). ***Insets***: T-b curves obtained by plotting median values of motility durations **(A)** and frequencies **(B)** against time. Quadratic functions (*y* = *a* + *bx* + *cx*^2^ with *a*, absolute term; *bx*, linear term; *cx*^2^, quadratic term) were fitted to the plots of motility durations, while linear functions (*y* = *ax* + *b* with *a*, slope and *b*, *y*-intercept) were fitted to the plots of motility frequencies. For the comparisons between groups (two-tailed *F* test, *α* = 0.0167 after Bonferroni correction) the respective *p* values are given.

After treatment with 5 mg/kg L-DOPA, the duration of head-shoulder motility (Figure 6A) was significantly reduced relative to vehicle in min 6–10 (*p* = 0.017) and min 21–25 (*p* = 0.010) as well as over the whole trial (*p* = 0.001). Differences relative to vehicle in min 11–15 (*p* = 0.035), min 16–20 (*p* = 0.038) and min 26–30 (*p = 0.045)* failed to reach significance after Bonferroni correction. Head-shoulder motility was also significantly shorter after with 10 mg/kg L-DOPA relative to vehicle in min 6–10, 11–15, 16–20 and 21–25 as well as from min 1–30 (0.0001 ≤ *p* ≤ 0.012). The differences relative to vehicle in min 1–5 with a *p* of 0.018 marginally failed to reach statistical significance after Bonferroni correction. Significant between-group differences were obtained from min 6.

T-b curves differed significantly between 5 mg/kg L-DOPA (quadratic fit; *a*, 40.3 ± 10.61; *b*, 3.42 ± 1.39; *c*; −0.13 ± 0.04) and vehicle (*a*: 34.0 ± 7.41; *b*, 8.43 ± 0.97; *c*; −0.25 ± 0.03; *p* = 0.0002) as well as between 10 mg/kg L-DOPA (*a*, 43.2 ± 11.18; *b*, 1.30 ± 1.46; *c*; −0.05 ± 0.04) and vehicle (*p* < 0.0001). After both L-DOPA doses, linear and quadratic terms fell short of linear and quadratic terms after vehicle suggesting a *slower* rate of increase of head-shoulder motility to a lower final level compared to vehicle.

Rats treated with 5 mg/kg L-DOPA moved their head and shoulders (Figure 6B) significantly less frequently throughout the whole testing time (*p* = 0.005). Differences relative to vehicle in min 11–15 (*p* = 0.035), min 16–20 (*p* = 0.029) and min 26–30 (*p* = 0.029) failed to reach significance after Bonferroni correction. Head and shoulders were also moved significantly less frequently in rats treated with 10 mg/kg L-DOPA compared to vehicle in min 1–5, 6–10, 11–15, 16–20 and 21–25 as well as over the whole trial (0.0001 ≤ *p* ≤ 0.005). Significant between-group differences were evident from the beginning.

T-b curves differed significantly between 5 mg/kg L-DOPA (linear fit; slope, −0.37 ± 0.12; *y*-intercept, 20.33 ± 2.40) and vehicle (slope, −0.45 ± 0.06; *y*-intercept, 26.80 ± 1.24; *p* = 0.007) as well as between 10 mg/kg L-DOPA (slope, −0.28 ± 06; *y*-intercept, 17.07 ± 1.19; vehicle: slope, −0.45 ± 0.06; *y*-intercept, 28.80 ± 1.24; *p* < 0.0001) and vehicle. The absolute values of the slopes after both L-DOPA doses were lower than slope after vehicle indicating that the frequency of head and shoulder motility decreased at a *slower* rate to a lower final level relative to vehicle.

#### Grooming

After 10 mg/kg L-DOPA, rats groomed for a significantly shorter time compared to animals treated with vehicle (*p* = 0.041; data not shown). There were no other significant between-group differences of grooming behavior.

### Correlation of motor/exploratory behaviors with DAT binding

After vehicle, DAT binding correlated positively with rearing frequency in min 21–25 (*r* = 0.546, *p* = 0.028) and duration of head-shoulder motility in min 21–25 (*r* = 0.586, *p* = 0.017). A marginal correlation was obtained between DAT binding and rearing duration in min 26–30 (*r* = 0.481, *p* = 0.058).

After 5 mg/kg L-DOPA, DAT binding correlated negatively with sitting frequency in min 11–15 (*r* = 0.635, *p* = 0.010) and positively with both duration (*r* = 0.523, *p* = 0.045) and frequency of head-shoulder motility in min 16–20 (*r* = 0.620, *p* = 0.013).

No significant correlations with behavioral parameters were observed after 10 mg/kg L-DOPA (0.072 ≤ *p* ≤ 0.892).

### PCA and CA

The PCA revealed that 79% of the cumulative variance could be explained by the first principal componenet (PC1), while PC2 to PC7 added up to 87%, 93%, 98% and 100%, respectively, of the cumulative variance (Table 1). The major eigenvector component of PC1 was “traveled distance” in min 1–30. The major eigenvector components of PC2 to PC7 were “traveled distance” in min 21–25, 6–10, 11–15 and 1–30, respectively (Table 1). The contribution of V_3_” to the individual principal components amounted to 0.

**Table 1:** **Main results of principal component analysis**.

PCs	% of VAR accounted for by PCs	Eigenvectors with the highest contribution to PCs	% of VAR accounted for by the eigenvectors with the highest contribution to PCs
PC1	78.9	traveled distance in min 1–30	10.6
PC2	8.3	traveled distance in min 26–30	48.3
PC3	5.9	traveled distance in min 26–30	50.0
PC4	2.7	traveled distance in min 6–10	70.4
PC5	2.2	traveled distance in min 26–30	54.6
PC6	1.1	traveled distance in min 11–15	17.6
PC7	0.04	traveled distance in min 1–30	10.6

*PC, principal component; VAR, variance*.

The CA evidenced that the data set could be grouped into four clusters comprising 19, 12, 14 and 7 rats. Table 2 lists the centroid means of all variables in cluster 1–4. Thereby, cluster 1 displayed the lowest V_3_”, and the shortest traveled distance, the shortest ambulation duration, the lowest ambulation frequency, the longest sitting duration, the highest sitting frequency, the shortest rearing duration, the lowest rearing frequency, the shortest duration of head-shoulder motility and the lowest frequency of head-shoulder motility in the individual time frames. In contrast, cluster 4 was characterized by the highest V_3_”, and the longest traveled distance, the longest ambulation duration, the highest ambulation frequency, the shortest sitting duration, the lowest sitting frequency, the highest rearing duration, the highest rearing frequency, the longest duration of head-shoulder motility and the highest frequency of head-shoulder motility in the individual time frames. In cluster 2 and 3 intermediate centroid means were obtained for each of these variables.

**Table 2:** **Centroid means of the individual variables obtained from cluster analysis**.

Variable	Cluster 1	Cluster 2	Cluster 3	Cluster 4
V3”	0.98	1.05	1.09	1.2
traveled distance in min 1–30 (cm)	4931	7474	9246	1234
traveled distance in min 1–5 (cm)	1960	2964	3481	3963
traveled distance in min 5–10 (cm)	782	1907	2125	2643
traveled distance in min 11–15 (cm)	865	1162	1335	1835
traveled distance in min 16–20 (cm)	396	567	7305	1546
traveled distance in min 21–55 (cm)	516	673	984	1528
traveled distance in min 26–30 (cm)	409	200	592	827
ambulation duration in min 1–30 (s)	228	284	396	453
ambulation duration in min 1–5 (s)	90	117	133	135
ambulation duration in min 6–10 (s)	46	67	95	98
ambulation duration in min 11–15 (s)	35	42	52	68
ambulation duration in min 16–20 (s)	14	23	30	63
ambulation duration in min 21–25 (s)	24	30	43	56
ambulation duration in min 26–30 (s)	3	2	6	8
ambulation frequency in min 1–30 (n)	43	67	90	123
ambulation frequency in min 1–5 (n)	18	30	34	41
ambulation frequency in min 6–10 (n)	7	17	21	26
ambulation frequency in min 11–15 (n)	7	10	13	18
ambulation frequency in min 16–20 (n)	3	4	7	15
ambulation frequency in min 21–25 (n)	5	5	8	15
ambulation frequency in min 26–30 (n)	18	10	33	32
sitting duration in min 1–30 (s)	1043	857	588	354
sitting duration in min 1–5 (s)	113	45	28	20
sitting duration in min 6–10 (s)	150	63	38	33
sitting duration in min 11–15 (s)	175	116	71	61
sitting duration in min 16–20 (s)	215	173	148	74
sitting duration in min 21–25 (s)	186	184	126	90
sitting duration in min 26–30 (s)	219	253	193	77
sitting frequency in min 1–30 (n)	56	45	44	35
sitting frequency in min 1–5 (n)	9	6	5	4
sitting frequency in min 6–10 (n)	9	8	6	5
sitting frequency in min 11–15 (n)	11	9	8	6
sitting frequency in min 16–20 (n)	10	10	10	7
sitting frequency in min 21–25 (n)	8	8	8	6
sitting frequency in min 26–30 (n)	7	8	9	6
rearing duration in min 1–30 (s)	79	130	201	284
rearing duration in min 1–5 (s)	32	57	70	86
rearing duration in min 6–10 (s)	16	38	50	67
rearing duration in min 11–15 (s)	11	19	33	44
rearing duration in min 16–20 (s)	6	10	11	38
rearing duration in min 21–25 (s)	11	7	25	34
rearing duration in min 26–30 (s	5	0	12	14
rearing frequency in min 1–30 (n)	22	40	54	84
rearing frequency in min 1–5 (n)	11	20	24	31
rearing frequency in min 6–10 (n)	4	11	13	20
rearing frequency in min 11–15 (n)	3	5	7, 5	12
rearing frequency in min 16–20 (n)	1	2	3	7
rearing frequency in min 21–25 (n)	2	2	5	10
rearing frequency in min 26–30 (n)	1	0	2	3
head-shoulder motility duration in min 1–30 (s)	354	363	410	499
head-shoulder motility duration in min 1–5 (s)	57	62	63	57
head-shoulder motility duration in min 6–10 (s)	65	89	90	83
head-shoulder motility duration in min 11–15 (s)	65	78	79	86
head-shoulder motility duration in min 16–20 (s)	56	59	75	103
head-shoulder motility duration in min 21–25 (s)	56	48	59	94
head-shoulder motility duration in min 26–30 (s)	43	33	50	74
head-shoulder motility frequency in min 1–30 (n)	79	84	98	123
head-shoulder motility frequency in min 1–5 (n)	14	195	22	25
head-shoulder motility frequency in min 6–10 (n)	13	19	21	26
head-shoulder motility frequency in min 11–15 (n)	15	17	18	20
head-shoulder motility frequency in min 16–20 (n)	12	13	15	21
head-shoulder motility frequency in min 21–25 (n)	11	11	13	19
head-shoulder motility frequency in min 26–30 (n)	9	8	11	14

## Discussion

### DAT binding

Challenge with 5 mg/kg L-DOPA/benserazide reduced DAT binding significantly by 23% relative to vehicle. The 14% reduction obtained after 10 mg/kg L-DOPA was not significant after Bonferroni correction. Cerebellar radioactivity concentrations did not differ between vehicle and L-DOPA challenges, indicating that no confounding effects were exerted on radioligand accumulation, e.g., by affecting cerebral perfusion.

Findings are consistent with previous findings obtained in PD patients (Guttman et al., 2001) and rats (Dresel et al., 1998; Sossi et al., 2010; Nikolaus et al., 2011b, 2013). They contradict, however, other findings in parkinsonian humans (Nurmi et al., 2000; Parkinson Study Group, 2002, 2004; Schillaci et al., 2005) and non-human primates (Laruelle et al., 1993; Fernagut et al., 2010). Sossi et al. (2010) were the first to demonstrate differential effects of L-DOPA on radioligand binding to the DAT dependent on the intactness or degeneration of the neostriatum in a rat model of PD. Their findings suggest that the extent of nigrostriatal neurodegeneration determines the number of available DAT binding sites, the capacity to synthesize and release DA contingent on administration of the precursor molecule and, consequently, also the extent, to which the exogeous radioligand may bind to the DAT. Accordingly, PD patients, who displayed a significant decrease of DAT binding after long-term treatment with L-DOPA had a mean Unified Parkinson’s Disease Rating Scale (UPDRS) score of 23.9 ± 6.0 (Guttman et al., 2001), whereas in studies, in which no alterations of DAT binding were observed, mean UPDRS scores were 17–47% higher (Schillaci et al., 2005: UPDRS, 35.1 ± 11.8; Nurmi et al., 2000: UPDRS, 31.0 ± 48.0; Parkinson Study Group, 2002: UPDRS, 30.6 ± 11.4; Parkinson Study Group, 2004: UPDRS, 28.0 ± 13.0). Also findings of reduced radioligand binding to the DAT as obtained in healthy rats in the present and in previous investigations (Dresel et al., 1998; Nikolaus et al., 2011b, 2013) are consistent with this assumption.

Since L-DOPA increases DA efflux (for review see Misu et al., 1996), the reduction of DAT binding may be interpreted in terms of competition between [^123^I]FP-CIT and endogenous DA. A further mechanism likely to occur at the presynaptic terminal in response to increased DA levels is the up-regulation of DAT binding sites (for review see Eriksen et al., 2010). As, however, DAT binding was found to be decreased after L-DOPA challenge, it can be concluded that at the time of [^123^I]FP-CIT administration DA levels had risen to an extent surmounting the capacity of DA reuptake by the available DAT binding sites. Thereby, it remains to be seen, whether and under which conditions the number of DAT binding sites in relation to synaptic DA concentrations can rise by such a degree that direct competition between [^123^I]FP-CIT and DA is avoided by DA reuptake in a sufficient quantity. If this can be the case, it was not detected with the L-DOPA doses and the time window between L-DOPA challenge and [^123^I]FP-CIT administration chosen in the present investigation.

Anesthetics inevitably influence the brain’s neurotransmitter systems. We employed ketamine, a compound, which has been found to increase the levels of DA, noradrenaline and other neurotransmitters in cortical and subcortical regions (for review see De Souza Silva et al., 2007; Müller et al., 2011). Since in our study L-DOPA- as well as saline-treated rats had undergone anesthesia prior to radioligand injection, it cannot be dismissed that DA levels were elevated by ketamine in both experimental conditions. In this case it may be expected, however, that the comparability of experimental conditions was preserved by the application of ketamine to both treatment groups. Ketamine may also have exerted different effects in L-DOPA and saline-treated rats, in which case, ketamine may have at least contributed to the L-DOPA-induced increase of striatal DA levels. Further investigations are needed, which explicitly address this question.

### Rat behavior—median differences and t-b curves

After both L-DOPA doses, the median sitting duration was higher compared to vehicle, while median duration and frequency of head-shoulder motility were lower. Findings are consistent with previous reports of reduced behavioral activity after administration of the DA precursor molecule in moderate doses (McDevitt and Setler, 1981).

As evidenced by Figures 2–6, the reductions of motor/exp­loratory behaviors and the increase of sitting after both L-DOPA doses relative to vehicle became significant around the second to third time frame (min 6–15). This coincides with the onset of measurable DA release, which in *in vivo* microdialysis studies was observed between min 5 and 15 after i.p. injection of L-DOPA (e.g., De Souza Silva et al., 1997a,b; Meissner et al., 2006) suggesting an association between the reduction of behavioral activity and the increased availability of endogenous DA in min 5–15 post-injection. Fifteen minutes after i.p. application of 25 (Meissner et al., 2006) and 50 mg/kg L-DOPA plus benserazide (De Souza Silva et al., 1997b), neostriatal DA concentrations reached 170% and 900%, respectively, of baseline levels. From this it may be concluded that also the lower L-DOPA doses employed in the present investigation elevated neostriatal DA to levels sufficient to decrease motor/exploratory behaviors and to compete with [^123^I]FP-CIT for DAT binding sites.

In the present study, the analysis of t-b curves for measures of sitting and head-shoulder motility yielded higher slopes and smaller constants in the linear and quadratic terms after both L-DOPA doses relative to vehicle indicating (1) a *faster* rate of increase of sitting duration to a higher final level; (2) a *slower* rate of increase of head-shoulder motility duration to a lower final level; and (3) a *slower* rate of decrease of head-shoulder motility frequency to a lower final level. In addition, the analysis of rearing duration curves yielded evidence of higher rate constants and lower plateau values after L-DOPA compared to vehicle suggesting a *faster* rate of decrease in rearing to a lower final level after L-DOPA relative to vehicle. These findings evidence a time-dependent decline of motor and exploratory behaviors in the open field. The waning of exploratory activities such as rearing and head-shoulder motility as well as the increase of sitting behavior within or between trials is generally considered to reflect behavioral habituation (for review see Leussis and Bolivar, 2006). Since these behavioral alterations are likely to be related to the L-DOPA-induced increase of endogenous DA levels in the neostriatum and DA is known to be involved in learning and memory (for review see Myhrer, 2003), the decline of behavioral markers of activity after L-DOPA also in the present investigation may be due to its action on the rate of behavioral habituation to a novel environment.

Behavioral alterations relative to vehicle were more pronounced after the higher dose of L-DOPA and related to more behavioral measures, which may be due to a larger effect on endogenous DA levels. This is not consistent with effects on DAT binding, as results indicated a biphasic response with a higher effect on DAT after the lower dose of L-DOPA. Biphasic regulatory actions of L-DOPA on DA and other neurotransmitters including noradrenaline and acetylcholine are widely known (for review see Misu et al., 1996) and raise the question, by which mechanisms the disparate effects on DA efflux and DAT binding are exerted. Dopamine release is modulated by a negative feedback loop established by DA acting upon presynaptic terminal autoreceptors of the inhibitory D_2/3_ receptor subtype (for review see Langer, 1997). Therefore, it may be that DA concentrations after 10 mg/kg L-DOPA at the time of radioligand application were lower compared to the 5 mg/kg dose, because in the 30 min time span between its application and the administration of the radioligand the higher dose may have promoted the release of DA in concentrations sufficient to activate feedback inhibition at the presynaptic terminal. If this was the case, neostriatal V_3_” values after higher L-DOPA doses can not be assumed to reflect DA levels at the time of data acquisition in the open field. This agrees with the *in vitro* finding of inhibited DA release after micromolar relative to nanomolar concentrations of L-DOPA (for review see Misu et al., 1996), and is also consistent with the slightly higher mean V_3_” values observed after 10 relative to 5 mg/kg L-DOPA in the present study (Figure 1). Further studies with higher doses of L-DOPA are required to shed further light on this matter.

### Correlation analysis, PCA and CA

After vehicle, DAT binding correlated positively with rearing duration (min 26–30) and frequency (min 21–25) as well as duration of head-shoulder motility (min 21–25). After 5 mg/kg L-DOPA, DAT binding correlated negatively with sitting frequency (min 11–15), and positively with both duration and frequency of head-shoulder motility (min 16–20). Correlation coefficients were low but significantly different from 0. No correlations with behavioral parameters were observed after 10 mg/kg L-DOPA.

The moderate association between DAT binding and parameters of motor and exploratory behavior was in agreement with the results of PCA and CA. The PCA revealed that the major part of the variance in the data set could be explained by the variable “traveled distance” in min 1–30. Cluster analysis, in addition, evidenced that the centroid means of V_3_” (0.98, 1.05, 1.09 and 1.2) were contiguous, but still could be related to varying means of motor and exploratory parameters. Thereby, in accordance with the results of correlation analysis, lower DAT binding was associated with a decrease of exploratory behaviors (rearing and head-shoulder motility) and an increase of sitting, whereas higher DAT binding was observed jointly with increased exploratory behaviors and decreased sitting. In addition to the findings obtained from correlation analysis, DAT binding could also be related to ambulation with lower DAT binding associated with a lower and higher DAT binding associated with a higher ambulatory activity. As the reduction of striatal DAT binding contingent on the administration of L-DOPA reflects the increased availability of DA, it follows that a higher amount of neostriatal DA decreased motor/exploratory behaviors and facilitated sitting. According to the results of correlation analysis, this relation holds for both vehicle and 5 mg/kg L-DOPA, which underlines the general importance of DA for either behavior.

It is, however, important to note that according to the PCA, the most relevant eigenvector component is the traveled distance but not the neostriatal V_3_”. This suggests a merely moderate association between motor/exploratory and neostriatal DA function, which is in agreement with previous evidence on the relevance of brain regions beyond the neostriatum (such as the hippocampus and the prefrontal cortex) for the unfolding of central L-DOPA action (Navailles et al., 2010). It is also consistent with the role assigned to neurotransmitters beyond DA (such as serotonin, acetylcholine and glutamate) for motor activity as well as habituation learning (e.g., Dringenberg et al., 1995; Carey et al., 1998; Schildein et al., 2002). In order to gain further information on this matter, future investigations are needed, which jointly assess the effect of serotonin, acetylcholine and glutamate action on DAT binding and parameters of motor and exploratory behaviors.

The modest role of the neostriatal V_3_” in explaining the variance of behavioral data may be also due to the time span between the acquisition of behavioral data (immediately after the administration of L-DOPA or vehicle) and DAT imaging (150 min post-challenge). It can not be dismissed that V_3_” values (and DA levels) at the time of *in vivo* imaging no longer corresponded to the DA levels at the time of data acquisition in the open field. Future investigations, in which behavior after L-DOPA is measured for a longer time than 30 min. Besides, DAT binding should be asseessed in different sets of animals at various time after the injection of the radioligand.

## Conclusions

The present complementary investigation of DAT binding and behavioral parameters yielded mean reductions of striatal DAT binding after L-DOPA, which reflected increased availability of endogenous DA. As L-DOPA-treated animals displayed less head-shoulder motility and more sitting than vehicle-treated animals, we hypothesize that the decrease of behavioral activity is associated with the increased availibility of DA. The analysis of t-b curves for sitting duration and duration as well as frequency of head-shoulder motility yielded higher slopes as well as smaller constants in the linear and quadratic terms after both L-DOPA doses relative to vehicle, which indicate (1) a *faster* rate of increase of sitting duration to a higher final level; (2) a *slower* rate of increase of head-shoulder motility duration to a lower final level; and (3) a *slower* rate of decrease of head-shoulder motility frequency to a lower final level. In addition, the analysis of rearing duration curves yielded higher rate constants and lower plateau values after L-DOPA compared to vehicle indicating a *faster* rate of decrease of rearing duration a lower final level of rearing duration after L-DOPA relative to vehicle. These findings suggest that decline of behavioral markers of activity after L-DOPA may be due to its action on the rate of behavioral habituation to a novel environment.

We here present a novel approach, which assesses between-group differences of behavioral alterations by fitting suitable models to the acquired data and statistically comparing t-b curves. The benefit of this method is the gaining of information on the temporal dynamics of behavior, which is not the case with the standard approach of creating dose-response curves relating alterations of behavior to dose but not to time. Importantly, the differences obtained between t-b curves are confirmed by the median differences between the individual time frames, which, on the one hand, served as an internal validation of the t-b approach. On the other hand, however, the calculation of median values in the individual time frames contributed information on the onset of behavioral alterations (and habituation learning), which may be fixed to min 6–15 after i.p. application of L-DOPA. Alterations of behavioral settings and procedures (e.g., application of higher or lower L-DOPA doses or a longer acquisition time of behavioral data) may be expected to lead to a further refinement of the individual t-b models. In addition, future investigations will reveal, to which extent the present approach of fitting and comparing t-b curves between treatments may be transferred to pharmacological challenges with other compounds affecting the DAergic system.

Since (motor) behavior in adult rats is also associated with D_1_ (for review see Undieh, 2010) and D_2_ receptor function (for review see Helmeste, 1983), the present investigations should be complemented by adding *in vivo* imaging of D_1_ and/or D_2_ receptor binding to the assessment of behavioral data. As the binding of exogenous radioligands may depend on the intactness of the neostriatum, joint investigation of DAT and/or D_2_ receptor binding and behaviors in 6-hydroxydopamine-lesioned animals should be added in order to gauge the effects of L-DOPA in relation to unlesioned rats. Common features of long-term treatment of with L-DOPA, in parkinsonian humans are movement disturbances including dyskinesias, on-off-phenomenon and wearing-off phenomenon (for review see Cenci et al., 2011). Therefore, further *in vivo* imaging studies on rats over a wider clinical dose range and, in particular, after long-term treatment with L-DOPA will help to unravel not only the regulatory mechanisms underlying DA synthesis and release but also the association of DAT and D_1_/D_2_ receptor binding with behavioral features.

## Conflict of interest statement

The authors declare that the research was conducted in the absence of any commercial or financial relationships that could be construed as a potential conflict of interest.
